# Rapid Transition from Inhaled Iloprost to Inhaled Treprostinil in Patients with Pulmonary Arterial Hypertension

**DOI:** 10.1111/1755-5922.12008

**Published:** 2013-01-09

**Authors:** Robert C Bourge, Victor F Tapson, Zeenat Safdar, Raymond L Benza, Richard N Channick, Erika B Rosenzweig, Shelley Shapiro, Richard James White, Christopher Shane McSwain, Stephen Karl Gotzkowsky, Andrew C Nelsen, Lewis J Rubin

**Affiliations:** 1 University of Alabama at BirminghamBirmingham, AL, USA; 2 Duke University Medical CenterDurham, NC, USA; 3 Baylor College of MedicineHouston, TX, USA; 4 Allegheny General HospitalPittsburgh, PA, USA; 5 Massachusetts General HospitalBoston, MA, USA; 6 Columbia Presbyterian Medical CenterNew York, NY, USA; 7 David Geffen UCLA School of Medicine, Greater Los Angeles VA Healthcare SystemLos Angeles, CA, USA; 8 University of Rochester Medical CenterRochester, NY, USA; 9 United Therapeutics Corp.Research Triangle Park, NC, USA; 10 UCSD Medical CenterSan Diego, CA, USA

**Keywords:** Iloprost, Inhaled, Pulmonary arterial hypertension, Quality of life, Treprostinil

## Abstract

**Background:**

Inhaled treprostinil is a prostacyclin analog approved for the treatment of pulmonary arterial hypertension (PAH) that may provide a more convenient treatment option for patients receiving inhaled iloprost while maintaining the clinical benefit of inhaled prostacyclin therapy.

**Aims:**

In this open-label safety study, 73 PAH patients were enrolled with primarily World Health Organization Class II (56%) or III (42%) symptoms. At baseline, most patients (93%) were receiving 5 μg of iloprost per dose but 38% of patients reported a dosing frequency below the labeled rate of 6–9 times daily. Patients initiated inhaled treprostinil at 3 breaths four times daily (qid) at the immediate next scheduled iloprost dose. The primary objective was to assess the safety of rapid transition from iloprost to inhaled treprostinil; clinical status and quality of life were also assessed.

**Results:**

Most patients (84%) achieved the target treprostinil dose of 9 breaths qid and remained on study until transition to commercial therapy (89%). The most frequent adverse events (AEs) were cough (74%), headache (44%), and nausea (30%), and five patients prematurely discontinued study drug due to AE (n = 3), disease progression (n = 1), or death (n = 1). At week 12, the time spent on daily treatment activities was reduced compared to baseline, with a mean total savings of 1.4 h per day. Improvements were also observed at week 12 for 6-min walk distance (+16.0; *P* < 0.001), N-terminal pro-B-type natriuretic peptide (−74 pg/mL; *P* = 0.001), and the Cambridge Pulmonary Hypertension Outcome Review (all domains *P* < 0.001).

**Conclusions:**

Pulmonary arterial hypertension patients can be safely transitioned from inhaled iloprost to inhaled treprostinil while maintaining clinical status.

## Introduction

Pulmonary arterial hypertension (PAH) is a rare, life-threatening disease of the pulmonary vasculature characterized by a progressive increase in pulmonary vascular resistance, and ultimately, right ventricular failure 1. Prostacyclin analogs mimic the effects of prostacyclin, an endogenous prostaglandin, to cause vasodilation of the pulmonary arterial bed and inhibition of platelet aggregation, and the therapeutic benefits of these therapies for the treatment of PAH are well established 2–7. Due to relatively short *in vivo* half-lives, prostacyclin analogs have been historically administered by either continuous intravenous or subcutaneous infusion. As such, the use of these therapies is complex and often challenging to administer 2. In recent years, inhaled prostacyclin analogs have emerged as attractive treatment options for PAH patients requiring prostacyclin therapy due to their relatively low incidence of systemic side effects, their ease of use compared to the parenteral therapies, and their ability to deliver vasodilatory effects directly to the lung vasculature reducing intrapulmonary shunting (V/Q mismatch) 2,8–10,11. In fact, the prostacyclin analogs iloprost (Ventavis®, Actelion Pharmaceuticals Ltd, Allschwil, Switzerland) and treprostinil (Tyvaso®, United Therapeutics Corp, Research Triangle Park, NC, USA) are both approved in the USA as inhaled therapies for the treatment of PAH 12,13.

While the mechanism of action of iloprost and treprostinil is similar, the *in vivo* pharmacokinetics (PK), and thus indicated treatment regimens, are different. Due to its relatively short half-life (20–30 min), the recommended administration schedule for inhaled iloprost is 6–9 doses (inhalations) per day with a minimum of 2 h between doses and a target maintenance dose of 5 μg per administration 12. Conversely, with an elimination half-life of approximately 4.5 h, the recommended dosing of inhaled treprostinil is four times per day (qid) with approximately 4 h between doses and a target maintenance dose of 9 breaths per treatment session 13. Given the more favorable administration schedule of inhaled treprostinil compared to inhaled iloprost, the objective of this study was to investigate the safety, efficacy, and quality of life (QoL) after rapid transition from inhaled iloprost therapy to inhaled treprostinil therapy in PAH patients.

## Methods

### Study Design

This study was a multicenter, prospective, open-label safety evaluation in PAH patients receiving stable iloprost therapy. The study was sponsored by United Therapeutics Corporation. Following institutional review board approval, all patients provided informed consent before any study-related assessments.

### Study Population

Eligible patients were between the age of 18 and 75 years with a diagnosis of idiopathic/hereditary PAH, PAH associated with collagen vascular disease or human immunodeficiency virus, or PAH associated with unrepaired or repaired congenital systemic-to-pulmonary shunt (repaired ≥5 years). Patients were required to have a baseline 6-min walk distance (6MWD) of ≥250 m and be receiving a stable dose of iloprost for at least 30 days prior to baseline. For patients receiving endothelin receptor antagonist (ERA) or PDE-5 inhibitor background therapy, a stable dose for those medications was required for 30 days prior to baseline. Women of childbearing potential were required to practice an acceptable method of birth control. Patients were considered ineligible if they were pregnant or nursing; had left-sided heart disease (World Health Organization [WHO] Group 2) or significant parenchymal lung disease (WHO Group 3); were receiving any investigational medication; or if they had changed or discontinued any PAH medication within 30 days.

### Study Objectives

The primary study objective was to evaluate the acute and long-term safety of inhaled treprostinil therapy following rapid transition from inhaled iloprost therapy. Secondary objectives were to evaluate the effect of inhaled treprostinil on 6MWD, Borg dyspnea index (BDI), plasma NT-proBNP, WHO functional class, and QoL in a group of previously stable iloprost patients.

### Study Drug

Following completion of all baseline study assessments, patients discontinued iloprost therapy during the baseline visit and initiated inhaled treprostinil at 3 breaths (6 μg/breath) qid. The initial dose of inhaled treprostinil occurred in the investigator clinic at the time of the patients' next scheduled dose of inhaled iloprost. The suggested treprostinil dose titration was an increase of one additional breath per dosing session every 3 days with a goal of 9 breaths qid within the first 3 weeks of treatment. If clinically indicated, investigators were allowed to increase to a maximum of 12 breaths qid. Prior to initiation of study drug, patients were trained on proper utilization of the OPTINEB® device (Nebu-Tec, Elsenfeld, Germany).

### Study Assessments

Baseline, week 6, week 12, and month 12 assessments included a physical examination, vital signs, 6MWD (BDI; immediately following 6MWD), WHO functional class, the Cambridge Pulmonary Hypertension Outcome Review (CAMPHOR) questionnaire 14, and clinical laboratory parameters including urine pregnancy screening, blood chemistries, hematology, coagulation times, and N-terminal probrain natriuretic peptide (NT-proBNP). All 6MWD and BDI assessments were conducted at peak drug concentrations (10–30 min postiloprost at baseline; 10–60 min post-treprostinil during treatment phase). Additionally, the drug administration activities questionnaire and the treatment satisfaction questionnaire for medicine (TSQM) 15 were conducted at baseline and week 12; the patient impression of change (PIC) assessment was conducted at week 12. For the drug administration activities questionnaire, patients were asked to provide information related to the daily administration and time requirements of inhaled iloprost (baseline) and inhaled treprostinil (week 12). In support of this analysis, patients were also given the option of completing a 7-day drug administration activities diary that recorded all time spent with the drug and/or device for the 7 days before baseline (on iloprost) and for the 7 days before week 12 assessments (on treprostinil.) Adverse events (AEs), including incidence, severity, and relatedness to study drug, were monitored throughout the study as were any changes in concomitant medications.

For analysis of inhaled treprostinil PK, blood samples were collected 10 min prior to dosing and 5, 10, 15, 20, 30, 45, 60, 90, 180, 270, and 360 min after dosing. Patients were eligible for PK analysis if they had been receiving inhaled treprostinil for at least 30 days and if they had been on a stable dose for at least 3 days. Plasma concentrations of treprostinil were determined using a validated method as described previously 16.

### Data Analysis

Numeric endpoints for postbaseline assessments were compared to baseline using a Wilcoxon signed rank test, and statistical significance was set at *P* < 0.05. Data are presented as observed case with no imputation for missing data. Analysis of secondary endpoints was descriptive in nature with no formal hypothesis testing. Statistical analysis was performed using SAS® software, version 9.2 (SAS Institute Inc., Cary, NC, USA). The database and all statistical outputs were retained by the sponsor, United Therapeutics Corporation. All authors had access to the data to enable confirmation of the findings. The authors assume full responsibility for the completeness and accuracy of the content of the manuscript.

## Results

### Patient Demographics and Disposition

Seventy-three patients were enrolled between December 2008 and December 2009 with a mean age of 49 years (range: 18–74). Patients were predominantly female (78%) with idiopathic/hereditary PAH (48%) and WHO functional class II/III (56/42%) symptoms (Table 1). Median baseline 6MWD was 378 m (interquartile range [IQR]: 330–452); median baseline plasma NT-proBNP concentration was 626 pg/mL (IQR: 222–1330). Most patients (59%) were receiving triple therapy (i.e., ERA, PDE-5 inhibitor, and iloprost).

**Table 1:** Baseline characteristics

Characteristic	N = 73
Age, year	49 (18–74)
Female	57 (78)
PAH etiology	
Idiopathic or hereditary	35 (48)
Collagen vascular disease	16 (22)
Other^a^	22 (30)
Background PAH therapy	
ERA only	19 (26)
PDE-5 inhibitor only	8 (11)
Both	43 (59)
None	3 (4)
WHO functional class	
I	1 (1)
II	41 (56)
III	31 (42)
IV	0 (0)
6MWD, m	378 (330–452)
NT-proBNP, pg/mL	626 (222–1330)

Values are mean (range) for age and median (interquartile range) for 6MWD and NT-proBNP. All other values are n (%). PAH, pulmonary arterial hypertension; ERA, endothelin receptor antagonist; PDE-5, phosphodiesterase type 5; WHO, World Health Organization; 6MWD, 6-min walk distance; NT-proBNP, N-terminal pro-B-type natriuretic peptide.

a Other PAH Etiology includes HIV infection (n = 3), repaired congenital shunt (n = 4), and unrepaired congenital shunt (n = 15).

Baseline iloprost usage is shown in Table 2. All patients were using the I-neb AAD® System (Philips Respironics, Pittsburg, PA, USA), and most patients (93%) were receiving 5.0 μg of iloprost per dose. Twenty-eight patients (38%) reported using iloprost less than the labeled frequency of 6–9 inhalations per day (Table 2). Seventy patients (96%) completed the week 12 assessments. Eight (11%) patients eventually discontinued the study drug due to AE (n = 3), withdrawn consent (n = 3), disease progression (n = 1), and death (n = 1) (Table 3). The majority of patients (n = 65) continued to receive treatment until the study was terminated by the sponsor (mean exposure = 32.4 weeks; range, 0.4–56.0), at which point most patients transitioned to commercial therapy.

**Table 2:** Inhaled prostacyclin dosing

Characteristic	N = 73
Baseline iloprost usage	
Dose	
2.5 µg	5 (7)
5.0 µg	68 (93)
Frequency	
<6× day	28 (38)
≥6× day	45 (62)
Inhaled treprostinil dosing	
Week 12 Dose	
<9 breaths	19 (26)
≥9 breaths	54 (74)
Were 9 breaths achieved?	
No	12 (16)
Yes	61 (84)
Time to reach 9 breaths (n = 61)	18 (7–22)

Values are n (%) and median (interquartile range) days.

**Table 3:** Summary of discontinuations and adverse events (AEs)

Characteristic	N = 73
Discontinued (overall)	8 (11)
AE	3 (4)
Withdrawn consent	3 (4)
Disease progression	1 (1)
Death	1 (1)
AEs (any event)	71 (97)
Cough	54 (74)
Headache	32 (44)
Nausea	22 (30)
Chest discomfort	12 (16)
Flushing	11 (15)
Nasopharyngitis	11 (15)
Upper respiratory tract infection	11 (15)
Dizziness	10 (14)
Palpitations	9 (12)
Throat irritation	9 (12)
Fatigue	8 (11)
Oropharyngeal pain	7 (10)
Productive cough	7 (10)

Values are n (%). Includes AEs occurring in at least 10% of patients. Mean exposure 32.4 weeks (range: 0.4–56.0).

### Dosing and Acute Tolerability

The mean (±SD) dose of inhaled treprostinil achieved was 8.8 ± 2.4, 8.9 ± 2.4, 9.3 ± 2.0, and 9.2 ± 1.4 breaths qid for week 6, week 12, month 6, and month 12, respectively. Most patients (84%) achieved the target dose of 9 breaths within approximately 18 days (Table 2). Analysis of AEs with onset during the first day of study drug dosing (cough [25%]; headache [11%]) and with onset during the first 5 days of study drug dosing (cough [38%], headache [27%] and nausea [8%]) was consistent with inhaled prostacyclin therapy and did not reveal any evidence of acute decompensation. There was one AE leading to discontinuation of study drug during the first 5 days of dosing that the individual investigator deemed “reasonably attributable” to study drug (psychotic disorder [day 3]).

### Safety

The most frequent AEs with inhaled treprostinil included cough (74%), headache (44%), and nausea (30%) (Table 3). Most AEs were mild or moderate in intensity; severe AEs were reported in 21 (29%) patients. Fifteen serious adverse events (SAEs) were reported in 10 (14%) patients, including two events each of pneumonia and worsening pulmonary hypertension. Most SAEs (10 [67%]) were considered by the investigator to be “not reasonably attributable” to study drug. Three (4%) patients prematurely discontinued study drug due to an AE, including two events of dyspnea and one event each of chest pain, cough, dysphonia, fluid retention, myocardial infarction, pulmonary hypertension, and psychotic disorder. One patient died during the course of the study due to disease progression (study day = 125). Although there were occasional transient changes in individual laboratory parameters during the study, there were no clinically significant, treatment-related changes in laboratory parameters following the transition to inhaled treprostinil.

### Efficacy

The median (IQR) change from baseline in 6MWD was increased at both week 6 (+9.5 m [−14 to 35]; n = 70; *P* = 0.008) and week 12 (+16.0 m [−8 to 39]; n = 68; *P* < 0.001), and this treatment effect appeared to be maintained through month 12 for patients with long-term data (Table 4). 6MWD improvements were associated with maintained or improved BDI values (Table 4). Compared with baseline, median (IQR) plasma concentrations of NT-proBNP were reduced at week 6 (−80 pg/mL [−376 to 50]; n = 69; *P* < 0.001) and week 12 (−74 pg/mL [−339 to 37]; n = 68; *P* = 0.001) and tended to be lower at month 12 for patients with long-term data (Table 4). WHO functional class was maintained or improved for the majority of patients at each postbaseline time point, with 96% of patients demonstrating maintained or improved functional status at both week 12 and month 12 (Table 4). Consistent with these changes in WHO functional class, clinical symptoms of PAH were also maintained or improved in the majority of patients.

**Table 4:** Change from baseline in 6MWD, NT-proBNP, and WHO functional class

	Week 6	Week 12	Month 6	Month 12
6MWD, m^a^	9.5 (−14 to 35)^d^	16.0 (−8 to 39)^e^	26.0 (−3 to 51)^e^	27.0 (−7 to 54)^d^
BDI^a^	−0.54 (0.20)^c^	−0.66 (0.22)^d^	−0.51 (0.27)^ns^	−1.06 (0.36)^d^
NT-proBNP, pg/mL^b^	−80 (−376 to 50)^e^	−74 (−339 to 37)^d^		−111 (−345 to 93)^ns^
WHO functional class				
Improved	4 (6)	6 (9)	11 (19)	7 (29)
Maintained	61 (87)	60 (87)	45 (78)	16 (67)
Worsened	5 (7)	3 (4)	2 (3)	1 (4)

Values presented as median (interquartile range), mean (SE), or n (%). 6MWD, 6-min walk distance; BDI, borg dyspnea index; NT-proBNP, N-terminal pro-B-type natriuretic peptide.

a 6MWD and BDI data for n = 70 (week 6), n = 68 (week 12), n = 55 (month 6), and n = 23 (month 12).

b NT-proBNP data for n = 69 (week 6), n = 68 (week 12), and n = 24 (month 12).

c *P* < 0.05.

d *P* < 0.01.

e *P* < 0.001.

ns, not significant.

### Quality of Life

**Figure 1:**
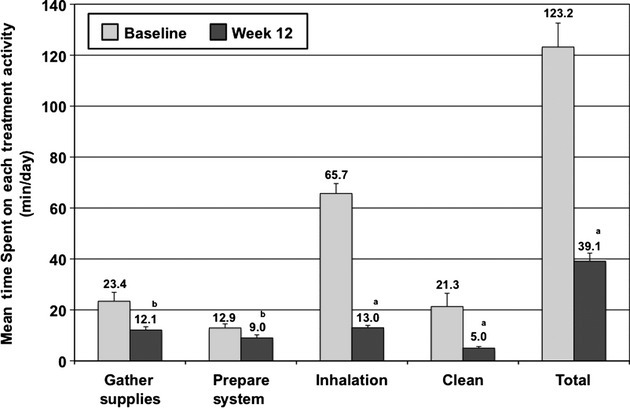
Time spent on daily treatment activities. The mean (±SE) time spent on each activity (min/day) is presented for baseline (iloprost; n = 70) and week 12 (inhaled treprostinil; n = 61). ^a^*P* < 0.001; ^b^*P* < 0.01.

The transition from iloprost to inhaled treprostinil reduced the time spent on daily treatment activities, with a 68% (*P* < 0.001) reduction in total time including reduced time spent gathering supplies (−48%; *P* = 0.004), preparing the treatment system (−30%; *P* = 0.007), inhalation (−80%; *P* < 0.001), and cleaning the treatment system (−77%; *P* < 0.001) (Figure 1). Across patients, the transition to inhaled treprostinil resulted in a mean total time saved of 1.4 h per day (39.1 min [week 12] vs. 123.2 min [baseline]). Treatment administration questionnaire data for the overall study population were supported by detailed, 7-day diary data (n = 16) that indicated a similar direction and magnitude of change in treatment administration times.

**Figure 2:**
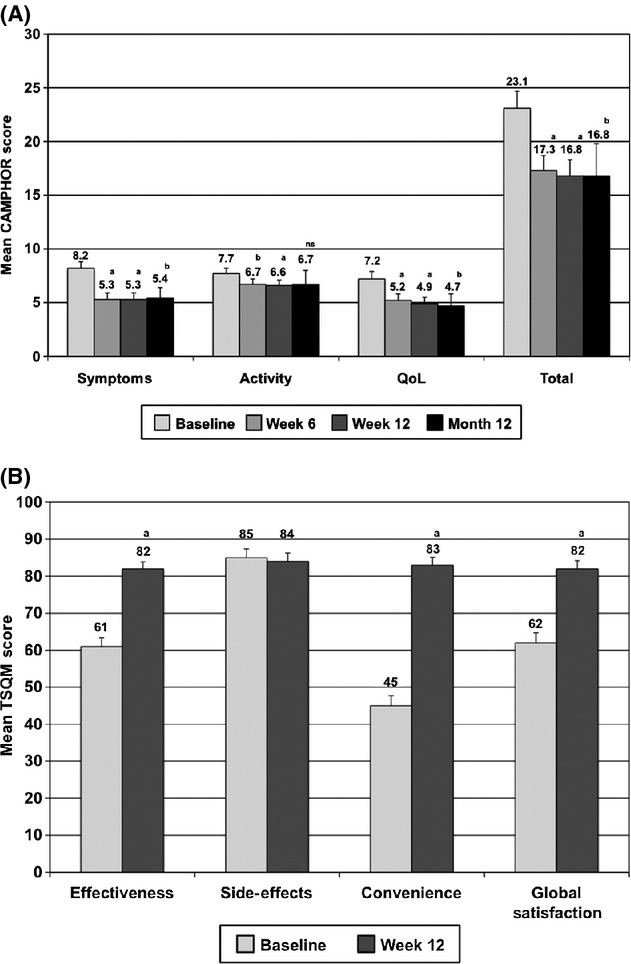
Cambridge pulmonary hypertension outcome review (CAMPHOR) and treatment satisfaction questionnaire for medicine (TSQM). (**A**) Mean (±SE) CAMPHOR scores presented for baseline (iloprost; n = 72), week 6 (inhaled treprostinil; n = 67), week 12 (inhaled treprostinil; n = 67), and month 12 (inhaled treprostinil; n = 24). ^a^*P* < 0.001; ^b^*P* < 0.05; ^ns^not significant. (**B**) The mean (±SE) TSQM score for each category is presented for baseline (iloprost; n = 72) and week 12 (inhaled treprostinil; n = 66). ^a^*P* < 0.001.

Improvements were observed for all domains of CAMPHOR at each assessment time, with the exception of the activity domain at month 12 (Figure 2A). CAMPHOR improvements tended to be maximal by week 6 and were largely maintained through 1 year for patients with long-term data. Analysis of the treatment satisfaction questionnaire (TSQM) for week 12 revealed improvements in effectiveness, convenience, and global satisfaction, with no change in side effects (Figure 2B). PIC data for week 12 versus baseline (n = 67) indicated that the majority of patients felt that their symptoms of PAH were much or somewhat better (73%; *P* < 0.001) and that the time spent on treatment administration was much or somewhat less (91%; *P* < 0.001). Overall, 94% (*P* < 0.001) of patients were much more or more satisfied with inhaled treprostinil therapy.

### Pharmacokinetics

**Figure 3:**
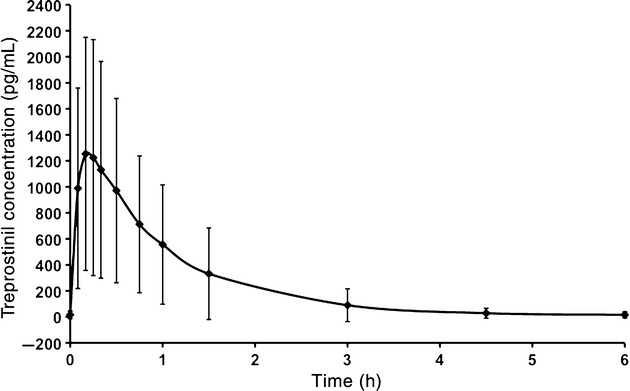
Mean (±SD) plasma treprostinil concentration versus time following administration of 54 μg of inhaled treprostinil (n = 11). Values are pg/mL.

Pharmacokinetics data were obtained in a cohort of 17 patients. The PK subpopulation was primarily female (82%) and Caucasian (94%), with a mean age of 51 years (range: 18–74). For patients receiving 9 breaths (54 μg) of inhaled treprostinil qid (n = 11), the geometric mean C_max_ was 1015.3 pg/mL with a high variability estimate (% coefficient of variation) of 118%. For AUC_(0-τ)_, the geometric mean was 993.6 h*pg/mL (151%) (Figure 3).

## Discussion

While inhaled iloprost provides an alternative to parenteral prostacyclin therapy, the relatively short half-life of the compound requires a frequent dosing schedule potentially limiting compliance and perhaps efficacy. Given the potential administration advantages of inhaled treprostinil with respect to dosing frequency and duration, this study examined the effects of rapid transition from inhaled iloprost to inhaled treprostinil in PAH patients. Overall, the results demonstrate that this transition was safe and well tolerated with no apparent loss of clinical status.

Common AEs reported were similar to those observed previously in the placebo-controlled trial for treprostinil and consistent with either the route of administration (cough and throat irritation) or well-known effects of prostacyclin therapy (headache, nausea, flushing, and dizziness) 8,10,17. The AE profile observed in the first few days after the transition was similar to that observed for the overall study period with no evidence of acute deterioration immediately following the transition to inhaled treprostinil. Overall, most AEs were mild to moderate in intensity and did not result in discontinuation of study drug.

Overall, the transition from inhaled iloprost to inhaled treprostinil resulted in a time savings of approximately 1.4 h per day. The data suggest that these time savings may have contributed to enhanced overall treatment satisfaction (TSQM), improved QoL (CAMPHOR), and a favorable PIC. While changes in 6MWD, NT-proBNP, and WHO functional class are well-established measures of PAH treatment efficacy, questionnaire-based analysis of QoL and treatment satisfaction following a switch in therapy have not been extensively investigated 18–20. Given the relative lack of studies employing these patient-reported metrics in a PAH population, the minimal important difference for each, and thus the clinical relevance of these findings, is unknown. Despite this limitation, the magnitude of change in CAMPHOR and TSQM following the transition to inhaled treprostinil compares favorably to that previously observed in both PAH and non-PAH populations 21–25.

Despite being clinically stable on study entry, 38% of patients reported iloprost usage below the labeled dose. Therefore, observed improvements in secondary endpoints such as 6MWD and NT-proBNP likely reflect compliance with the labeled dosing frequency rather than specific differences between the molecules. Together, these data suggest that the treatment administration advantages of treprostinil may have allowed for more study patients to better reach their target prostacyclin exposure. Importantly, a higher concentration of inhaled iloprost (20 μg/mL) was approved for use during the course of this trial, with a goal of reducing treatment time 12. In fact, in a retrospective analysis of RESPIRE registry patients (n = 11), the 20 μg/mL iloprost concentration reduced treatment time by 56% 26. While it is unknown how many patients in this study were receiving this higher iloprost concentration at baseline, it is possible that had this treatment option been available at the start of the study, the patient-reported differences in treatment administration time seen in this study would have been reduced.

This study provides the first analysis of the PK of inhaled treprostinil in PAH patients following titration to the recommended maintenance dose of 54 μg qid. While the sample size is limited, the observed values for C_max_ (1015 pg/mL) and AUC_(0-τ)_ (994 h*pg/mL) are consistent with those previously observed in healthy volunteers and PAH patients 13,27,28. Based on the C_max_ observed in this study, the peak plasma concentration achieved with 54 µg qid of inhaled treprostinil in PAH patients is roughly comparable to the steady-state plasma levels seen with continuous infusion (subcutaneous or intravenous) of 10 ng/kg/min in healthy volunteers 16.

### Limitations

The conclusions drawn from this study are limited by the fact that this was an open-label trial with no placebo or active comparator; however, a blinded trial would have partially defeated the rationale of this observational study, which was to assess the safety and tolerability of transition from a 6 to 9 times daily therapy to a qid therapy. In addition to these requisite differences in therapy administration frequency, differences in nebulizer device also prevented the implementation of a blinded study design. This open-label design may have increased the chances of enrolling patients who were dissatisfied with their current iloprost therapy (i.e., selection bias). It is unknown whether patients receiving the higher iloprost concentration at baseline would have demonstrated similar changes in treatment administration time, QoL, and efficacy. Given that patients were transitioned to inhaled treprostinil at baseline, there was no collection of safety data while patients were receiving iloprost, thus preventing any direct comparison of the relative safety profiles across the two therapies. Patient-reported QoL and treatment administration time questionnaire data are inherently subjective, and the minimally important difference for these metrics has not been established for PAH patients. As such, the clinical relevance of the observed changes is unknown and the data should be interpreted with caution. Long-term data beyond week 12 are limited by a relatively small sample size and may be affected by a completer bias that would not account for patients who may have discontinued the trial for reasons such as treatment dissatisfaction. Given these concerns, interpretations of data beyond week 12 should be limited.

## Conclusions

In summary, these data indicate that rapid transition from inhaled iloprost to inhaled treprostinil in PAH patients is safe with no apparent loss of clinical efficacy. These data suggest that the administration advantages of inhaled treprostinil allowed for a reduction in total treatment preparation and administration times per day that may have resulted in increased dosing compliance, more appropriate prostacyclin exposures, and possibly enhanced therapeutic benefit.
